# COVID-19 Misinformation on Social Media: A Scoping Review

**DOI:** 10.7759/cureus.24601

**Published:** 2022-04-29

**Authors:** Andrew M Joseph, Virginia Fernandez, Sophia Kritzman, Isabel Eaddy, Olivia M Cook, Sarah Lambros, Cesar E Jara Silva, Daryl Arguelles, Christy Abraham, Noelle Dorgham, Zachary A Gilbert, Lindsey Chacko, Ram J Hirpara, Bindu S Mayi, Robin J Jacobs

**Affiliations:** 1 Medical School, Dr. Kiran C. Patel College of Osteopathic Medicine, Nova Southeastern University, Fort Lauderdale, USA; 2 Basic Sciences, Dr. Kiran C. Patel College of Osteopathic Medicine, Nova Southeastern University, Fort Lauderdale, USA; 3 Medical and Behavioral Research; Health Informatics; Medical Education, Nova Southeastern University, Fort Lauderdale, USA

**Keywords:** coronavirus, facebook, youtube, twitter, social media, sars-cov-2, misinformation, disinformation, pandemic, covid-19

## Abstract

Social media allows for easy access and sharing of information in real-time. Since the beginning of the coronavirus disease (COVID-19) pandemic, social media has been used as a tool for public health officials to spread valuable information. However, many Internet users have also used it to spread misinformation, commonly referred to as “fake news.” The spread of misinformation can lead to detrimental effects on the infrastructure of healthcare and society. The purpose of this scoping review was to identify the sources and impact of COVID-19 misinformation on social media and examine potential strategies for limiting the spread of misinformation. A systemized search of PubMed, Embase, and Web of Science electronic databases using search terms relevant to the COVID-19 pandemic, social media, misinformation, or disinformation was conducted. Identified titles and abstracts were screened to select original reports and cross-checked for duplications. Using both inclusion and exclusion criteria, results from the initial literature search were screened by independent reviewers. After quality assessment and screening for relevance, 20 articles were included in the final review. The following three themes emerged: (1) sources of misinformation, (2) impact of misinformation, and (3) strategies to limit misinformation about COVID-19 on social media. Misinformation was commonly shared on social media platforms such as Twitter, YouTube, Facebook, messaging applications, and personal websites. The utilization of social media for the dissemination of evidence-based information was shown to be beneficial in combating misinformation. The evidence suggests that both individual websites and social media networks play a role in the spread of COVID-19 misinformation. This practice may potentially exacerbate the severity of the pandemic, create mistrust in public health experts, and impact physical and mental health. Efforts to limit and prevent misinformation require interdisciplinary, multilevel approaches involving government and public health agencies, social media corporations, and social influencers.

## Introduction and background

Social media is defined as “forms of electronic communication (such as websites for social networking and microblogging) through which users create online communities to share information, ideas, personal messages, and other content (e.g., videos) [1]. Between 2021 and 2022, there were approximately four billion social media users worldwide, with the largest social media networks being Facebook, Instagram, Twitter, YouTube, and TikTok [2]. With the advent of social media, information has become easier to access, leading to the creation of an “infodemic” or abundance of knowledge. If used responsibly, these platforms can assist in rapidly supplying the public with newfound information, scientific discoveries, and diagnostic and treatment protocols [3]. Furthermore, it can help compare the various approaches countries around the world are implementing to curb the spread and severity of coronavirus disease (COVID-19) [3]. However, information disseminated may not be reliable or trustworthy and has the potential to cause undue stress, particularly during public health emergencies [4].

Since COVID-19 was declared a pandemic in March 2020, social media has given health officials, governments, and civilians an open platform to share information publicly, easily, and speedily [5]. In this regard, social media platforms have created a constant and proverbial “tug-of-war” between the efforts of health officials to disseminate evidence-based scientific information to mitigate the effects of the pandemic and the unmonitored spread of misinformation by social media users [5].

Misinformation, colloquially referred to as “fake news,” is defined as false, inaccurate information that is communicated regardless of an intention to deceive [6]. Social media users, some of whom are driven by self-promotion or entertainment, may be less likely to fact-check information before sharing it [7]. Computer algorithms used by social media platforms tend to provide content that is tailored for like-minded individuals, which may reinforce their radical ideology [6]. In turn, this phenomenon may lead to conspiracy theories and a lack of trust in health officials, healthcare workers, and healthcare mandates [8]. Additionally, this excessive exposure to sensationalized social and other media reporting of disasters may be associated with poorer population mental health outcomes [9,10].

As more information circulated regarding COVID-19, many social media users took to their respective platforms to share a myriad of conspiracy theories [11]. These conspiracies can greatly impact an individual’s behavior and undermine the overall efficacy of a government’s implemented regulations regarding COVID-19 [12]. One such theory was related to the concurrence of the COVID-19 pandemic and the release of the wireless network [11]. Users postulated that the 5G network was the culprit behind the rampant spread of COVID-19 [11]. The spread of certain conspiracy theories including rumors of ingesting chloroquine, cow urine, or hot water as possible cures [13,14]. There even have been cases involving ibuprofen or other anti-inflammatory drugs because of the erroneous idea that they could increase the chances of getting infected with COVID-19 [15,16]. Ultimately, these conspiracy theories and misinformation can lead to erroneous beliefs and attitudes about the pandemic [13,14]. As a result, preventative measures and recommendations given by public health officials to halt the spread of the severe acute respiratory syndrome coronavirus 2 (SARS-CoV-2) virus, including the use of masks and vaccinations, are disregarded [8]. Questioning the effectiveness of masks and the vaccine, along with a disregard for social distance mandates, may lead to life-threatening attitudes about the virus [13]. Furthermore, this may lead to overall doubt about the healthcare system as a whole [13].

Public figures, celebrities, and social influencers have also influenced how the public receives and interprets information by popularizing both conspiracy theories as well as efforts to halt the pandemic, including amplifying current healthcare guidelines and pushing the narrative to “flatten the curve” [17]. To further combat misinformation and discredit myths about COVID-19, the World Health Organization (WHO) has taken to social media to release shareable informational graphics [18,19]. Nevertheless, the dissemination of misinformation on social media about the COVID-19 pandemic may hinder efforts to slow down or stop the spread of the virus [17]. Stopping this pandemic with increased public health measures and social media panic, fueled by misinformation and disinformation, will be of the utmost importance in the fight against COVID-19 [20]. The purpose of this study was to identify misinformation or disinformation spread worldwide through various sources of social media and examine potential strategies for addressing this phenomenon.

## Review

A computerized search was performed to identify the sources and impact of COVID-19 misinformation on social media. The databases PubMed, Embase, and Web of Science were utilized with search terms related to the COVID-19 pandemic, social media, and misinformation.

Search strategy

Inclusion and exclusion criteria were established prior to performing the review. Articles were included if they (1) were in the English language, (2) were published in 2020, and (3) included an abstract containing the keywords COVID-19, misinformation, disinformation, and social media. Book reviews, correspondence, editorials, podcasts, radio and television segments, newspaper articles, print media, letters, notes, conference abstracts, short surveys, erratum, conference papers, and book chapters were excluded.

Identification of studies

An electronic search of PubMed, Embase, and Web of Science was performed to identify misinformation spread of COVID-19 on social media. An initial Boolean and keyword search that included “COVID 19” OR “COVID-19” OR “coronavirus” OR “SARS-CoV-2” AND “misinform*” OR “disinform*” OR “false” OR “rumor” OR “inaccuracy” AND “social media” OR “Twitter” OR “Facebook” OR “website” OR “Instagram” OR “Snapchat” OR “TikTok” OR “WhatsApp” was conducted.

Data extraction

**Figure 1:**
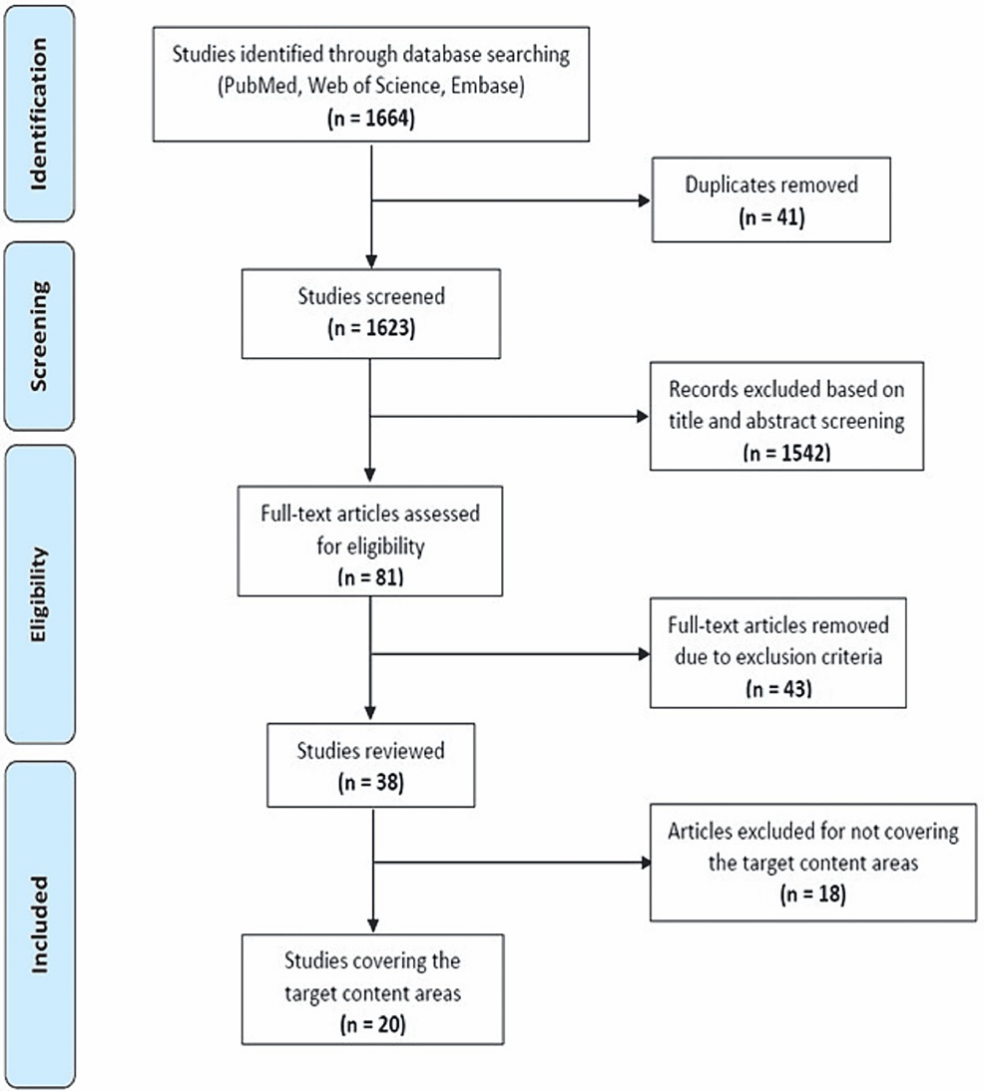
PRISMA flow diagram. PRISMA: Preferred Reporting Items for Systematic Reviews and Meta-Analyses

The Boolean and keyword search resulted in 1,664 articles, and 41 duplicates were removed. Of the 1,623 articles that remained, 1,542 articles were removed due to either (1) the abstract not including all keywords (COVID-19, misinformation, disinformation, and social media) or (2) being a book review, correspondence, editorial, podcast, radio, television, newspaper, print media, letter, note, conference abstract, short survey, erratum, conference paper, or chapter. Additionally, any article not written in English or not published in 2020 was removed. The remaining 81 full-text articles were assessed by two readers based on the inclusion and exclusion criteria, and a total of 38 articles were found to be eligible. Upon further screening, 18 did not meet our criteria for the content we set out to assess, leaving 20 articles for this review (see Figure 1).

Results

The search yielded a total of 1,664 articles using the predetermined search criteria. After 41 duplicates were removed, book reviews, correspondence, editorials, podcasts, radio, TV, newspaper, print media, letters, notes, conference abstracts, short surveys, erratum, conference papers, or chapters were excluded. An additional 43 articles were deemed unsuitable for inclusion as they were not published in English, published in 2020, or their abstract did not contain the keywords “COVID-19,” “misinformation/disinformation,” and “social media.” A final 17 articles were removed because their content could not be extrapolated to the general populace, resulting in a total of 20 articles for the final analysis.

Table 1 reports the characteristics of the studies included in the review, which included eight cross-sectional studies, five qualitative content analyses, four discussion articles, one descriptive analysis of quantitative data, one narrative analysis, and one systematic literature review. These studies were generated from Bangladesh (n = 1), Ukraine (n = 1), United Kingdom (n = 2), United States (n = 3), Zimbabwe (n = 1), globally in which multiple languages were spoken (n = 5), and globally in which only English was spoken (n = 7).

**Table 1:** A summary of the general characteristics of included studies. COVID-19: coronavirus disease 2019

Study citation details	Study design	Purpose of the study	Measures	Key findings
Strategies to limit misinformation				
Ahmed et al., 2020 [11]	Social network and content analysis	Develop an understanding of the drivers of the 5G COVID-19 conspiracy theory and strategies to deal with this misinformation	Social network analysis (betweenness centrality score, network group, 7 in total). Group 1: isolates group, Group 2: broadcast group, Group 3: activism account, Group 4: user analysis (account description, betweenness centrality score, follower count, network group), Group 5: influential web sources (website, web domains), Group 6: content analysis (theme, number of tweets)	Humor effect noted when discussing conspiracy theory. Influential websites include YouTube, Infowars, and a website dedicated to linking 5G to COVID-19. 34.8% (n = 81/233) of individual tweets contained views that 5G and COVID-19 were linked. Several ways to mitigate the spread of conspiracy theories: If the accounts set up to spread misinformation were taken down faster. An authority figure with a sizeable following could have tweeted messages against the conspiracy theory and urged other users that the best way to deal with it is to not comment on, retweet, or link bait using the hashtag. The fight against misinformation should take place on the platform where it arises. It is important to analyze the context of the fake news and why it is spreading
Kouzy et al., 2020 [28]	Content analysof twitter hashtags	Analyze the magnitude of misinformation that is being spread on Twitter regarding the coronavirus pandemic	Account type, informal individual/group; business/NGO/government news outlet/journalist; and healthcare/public health/medical. verification status; tweet tone, Serious, humorous, opinions; tweet’s degree of accuracy; correct information regarding COVID-19, misinformation regarding COVID-19; unverifiable information regarding COVID-19	The number of followers per account, number of likes per tweet, and the number of retweets per tweet were not associated with any significant difference in terms of unverifiable information rates. Accounts with a higher number of followers had fewer tweets with misinformation. The search term “Corona” was associated with the highest rate of unverifiable information, while the search terms “COVID-19” and “#coronavirusoutbreak” had the lowest levels of unverifiable information
Naeem et al., 2021 [13]	Content analysis	To identify the types and sources of COVID-19 misinformation	Sources of false news claims. Spread of fake news between January and April 2020. Types of misleading information. Relational analysis of co-occurrence of interrelated terms	The COVID-19 “infodemic” is full of false claims, half-baked conspiracy theories, and pseudoscientific therapies regarding the diagnosis, treatment, prevention, origin, and spread of the virus
Tangcharoensathien et al., 2020 [27]	Narrative analysis	To summarize the proceedings and outcomes of the consultation and recommendations for further action by the WHO, its member states, and other stakeholders	Providing coordination and development of guidelines and frameworks to reach all communities and vulnerable groups. Specific practical actions such as tailoring messages to specific audiences; developing and applying research methods to understand the “infodemic” at the level of information flows, populations, individuals, and communities; and analyzing the adherence to, and impact of, public health measures	Interventions and messages must be based on science and evidence and reach citizens to enable them to make informed decisions on how to protect themselves and their communities in a health emergency knowledge should be translated into actionable behavior-change messages, presented in ways that are understood by and accessible to all individuals in all parts of all societies. Governments should reach out to key communities to ensure their concerns and information needs are understood, tailoring advice and messages to address the audiences they represent. Strategic partnerships should be formed across all sectors to strengthen the analysis and amplification of information impact health authorities should ensure that recommended actions are supported with evidence that communities can read to understand
Bowles et al., 2020 [32]	Survey with list experiment	Examine how information from trusted social media sources can shape knowledge and behavior targeting widespread COVID-19 misinformation and mistrust	Effects of exposure to WhatsApp messages targeting misinformation: measured responses to factual questions (knowledge) and behavior changes related to the WhatsApp messages; treatment/control groups	Significant increase in knowledge about COVID-19 was found after exposure to WhatsApp messages from trusted sources. Harmful behavior (not abiding to guidelines) decreased by 30% after exposure to messages. Citizens were most likely to trust an international organization first, followed by local NGOs or CSOs and news sources
Baniya et al., 2020 [30]	Review article	Discuss the huge role social media plays in spreading information and misinformation, and how it can be used by professionals to better spread critical information during public health crises	Struggles of social media platforms to control misinformation	Need a new approach to tackle misinformation on social media, suggest standards for validating the professional status of people, and a way to display the expertise of said person on social media so people can trust their information
Baker et al., 2020 [29]	Review article	Discuss dissemination of information on social media, analyze how companies have tried to combat its spread, and the limitations involved in monitoring online content for misinformation	Comparing effectiveness of the strategies developed by social media platforms to combat misinformation	Difficult to flag misinformation in real time as “harmful” when not much is confirmed about the evolving pandemic. Flagging information as “harmful” can be perceived differently by people. Misinformation should be tagged in such a way to indicate that it goes against public health officials, allowing for more transparency
Malhotra et., 2020 [24]	Research article	Discuss the role of mobile instant messaging services like WhatsApp on the spread of misinformation	Culture dynamics and relational correction regarding misinformation	Need a micro-level approach to tackling misinformation and focus on culturally specific interactions between individuals to correct misinformation
Mututwa et al., 2020 [17]	Qualitative content analysis (QCA)	Explore how selected international celebrities appropriated their Twitter micro-blogging pages to announce their COVID-19 infection	In QCA, content embraces all appropriate data sources beyond the text such as images, videos, audio, graphics, and symbols	Social influencers including heads of state (e.g., President of the United States) amplified approved health guidelines to reduce the spread of COVID-19 by popularizing it as “flattening the curve.”
Lopez, 2020 [33]	Article discussion	Discuss the role that social media of local health departments can have to quickly and effectively disseminate factual information to a local population	Effectiveness of risk communication plans depending on the resiliency of communities	Social media should be used by local health departments to disseminate information rapidly and effectively to their respective communities. Eight out of ten consumers of social media news say they have been following news of the outbreak closely, with a majority of those consumers being exposed to some misinformation about coronavirus. Risk communication plans help build resilient communities and long-lasting emergency response systems
Pennycook et al., 2020 [31]	Research study using surveys conducted online using Lucid	Analyze how the “accuracy nudge” with social media headlines affected an individual’s potential to share both true or false information on social media platforms	In the first study, subjects were given 30 articles, 15 with accurate information and 15 with false information and were asked to identify if they were accurate or not, and if they would share that information on their social media profile. In the second study, they were only shown headlines and asked if they would share it on social media	In the first study, individuals who were more likely to rely on their intuitions and who were lower in basic scientific knowledge were worse at discerning between true and false content (in terms of both accuracy and sharing decisions). In the second study, participants were first asked to judge the accuracy of the news headline before being asked whether they would share it, which nearly tripled participants’ level of discernment between sharing true and sharing false headlines. People were distracted from accuracy by more fundamental aspects of the social media context, which plays an important role in the sharing of misinformation online. Nudging people to think about accuracy of information before sharing it is a simple way to improve choices about what to share on social media
Sources of misinformation				
Islam et al., 2020 [4]	Descriptive analysis of quantitative data	A study of 2,311 online reports of rumors, stigma, and COVID-19 conspiracy theories in 25 languages from 87 countries between December 31, 2019, and April 5, 2020	Category of information, accuracy of information, and (graphical) distribution of data	Online platforms studied included Facebook, Twitter, agency websites, and online newspapers. Most claims were related to illness, transmission, and mortality (24%), control measures (21%), treatment and cure (19%). 82% of claims were false misinformation being fueled by rumors stigma and conspiracy theories were found to have potentially serious implications on the individual and community (e.g., Asian biases)
Allington et al., 2020 [22]	Three online questionnaire surveys of social media use, conspiracy beliefs, and health-protective behaviors regarding COVID-19 among UK residents	Examine the relationship between COVID-19 conspiracy beliefs, social media use, and health-protective behaviors	Conspiracy belief, health-protective behavior, and information source	Positive relationship between holding one or more conspiracy beliefs and preference for social media over legacy media as a general source of information. Very strong negative relationship between holding one or more conspiracy beliefs and following all health-protective behaviors. YouTube had the strongest association with conspiracy beliefs, followed by Facebook. COVID-19 conspiracy beliefs are more likely to be held by younger respondents. Health-protective behavior was associated with both older age and female gender
Li et al., 2020 [14]	Cross-sectional study	1. Evaluate the accuracy, usability, and quality of the most viewed YouTube videos on COVID-19. 2. Propose recommendations for professional organizations to make use YouTube and expand the delivery of accurate information regarding COVID-19	Video characteristics, source of videos, factual vs non-factual videos	Over 25% of YouTube’s most viewed English videos contained non-factual or misleading information, reaching over 62 million views and nearly 25% of total viewership. Lack of access of professional and statistical reports may not be as appealing or accessible to the public
Lobato et al., 2020 [23]	Online preregistered exploratory survey	Assess whether patterns of individual differences in political orientation, social dominance orientation, traditionalism, conspiracy ideation, or attitudes about science predict willingness to share misinformation about COVID-19 pandemic online	296 participants via Amazon Mechanical Turk; Snopes & FactCheck to ask about willingness to share statements about severity/spread of COVID, treatment/prevention of COVID, COVID-19 conspiracies, and miscellaneous incorrect information on their social media; participants were informed all statements were false after completing survey	Individuals more aligned with liberal policy and less oriented with social dominance were less likely to spread conspiracy-themed misinformation on social media. Individuals who were high in social dominance and low in traditionalism were less likely to spread misinformation about the severity/spread of COVID-19, but more willing to spread conspiracy themed misinformation and miscellaneous information
Yüce et al., 2020 [21]	Cross-sectional study	To evaluate the quality of dentistry-related medical information about COVID-19 on YouTube as educational resources for dental practitioners	Quality of YouTube videos, ranked 1-5, with internal criteria created by researchers	Only two out of 55 videos that were reviewed contained high-quality information and content on reducing COVID-19 transmission in dental practices. Health professionals should be more active in providing educative information on social media during global disease outbreaks
Patel et al., 2020 [25]	Systematic literature review	Examine reports of disinformation surrounding health crisis communication in Ukraine during the COVID-19 response	News articles, technical reports, policy briefs, and peer-review publications that include data on COVID-19 in Ukraine and the messaging about it	34% of the included publications were published in March 2020, which was a 500% increase from the number of the identified publications published in January 2020. Recommended to increase transparency with verified health crisis messaging and address the leadership gap in reliable regional information about COVID-19 resources and support in Ukraine
Impact of misinformation on health				
Li et al., 2020 [26]	Content analysis	Examine stigma communication about COVID-19 on Twitter in the early stages of the outbreak and explore whether the presence of misinformation and conspiracy theories in COVID-19-related tweets is associated with the presence of COVID-19 stigma content	A total of 155,353 unique COVID-19-related tweets posted between December 31, 2019, and March 13, 2020, were identified, from which 7,000 tweets were randomly selected for manual coding	The peril of COVID-19 was mentioned the most often, followed by mark, responsibility, and group labeling content. Tweets with conspiracy theories were more likely to include group labeling and responsibility information, but less likely to mention COVID-19 peril. Public health agencies should be aware of the unintentional stigmatization of COVID-19 in public health messages and the urgency to engage and educate the public about the facts of COVID-19
Islam et al., 2020 [4]	Survey, cross-sectional study	To investigate how motivational factors and personal attributes influence social media fatigue and the sharing of unverified information during the COVID-19 pandemic among young adults in Bangladesh	Multivariate assumptions	People driven by self-promotion and entertainment, and those suffering from deficient self-regulation, are more likely to share unverified information. Exploration and religiosity correlated negatively with the sharing of unverified information
Romer et al., 2020 [8]	National probability survey of US adults (N = 1,050) was conducted in the latter half of March 2020 and a follow-up with 840 of the same individuals in July 2020	To see if conspiracy theories about COVID-19 on social media would negatively affect preventative measures taken by people	Adoption of preventive measures recommended by public health authorities, vaccination intentions, conspiracy beliefs, perceptions of threat, belief about the safety of vaccines, political ideology, and media exposure patterns	Belief in COVID-19-related conspiracy theories was inversely related to some factors, including perceived pandemic threat, taking preventative actions like mask-wearing, and perceived safety of and intention to obtain vaccination. Although adopting preventive behaviors was predicted by political ideology and conservative media reliance, vaccination intentions were less related to political ideology

The following three major themes emerged from the articles selected for this review: (1) sources of COVID-19 misinformation on social media, (2) impact of COVID-19 misinformation on social media, and (3) strategies to limit COVID-19 misinformation on social media.

Sources of Misinformation

Among the 20 articles, six focused on describing the source of misinformation. The most common social media platforms used included Twitter, YouTube, and Facebook, as well as websites dedicated to spreading COVID-19 misinformation. One article provided a global analysis of the rumors, stigma, and conspiracy theories across 25 languages from 87 countries, where 82% of 2,311 reports acquired from Twitter, Facebook, and online newspaper claims were false [4]. Two cross-sectional studies measured the quality and accuracy of the information about COVID-19 on YouTube [14,21]. On YouTube, 25% of the most viewed English videos involved non-factual or misleading information, and fewer than 4% of videos provided high-quality content on reducing COVID-19 transmission, specifically in dental practices [21].

Three studies conducted surveys to analyze factors contributing to the spread of misinformation on social media [6,22,23]. One study revealed that people driven by self-promotion and entertainment and those suffering from deficient self-regulation are more likely to share unsupported information [6]. A strong negative relationship was found between holding one or more conspiracy beliefs and following all health-protective behaviors. Among social media platforms, YouTube had the strongest association with conspiracy beliefs, followed by Facebook [22]. Individuals more aligned with liberal policy and less focused on social dominance were less likely to spread conspiracy-themed misinformation on social media as opposed to groups more oriented to social dominance who shared more beliefs on conspiracy theories and less on the severity and spread of COVID-19 [23]. The fight against misinformation should take place on the platform where it arises. It is important to analyze the context of fake news and why it is spreading [23]. Other sources of misinformation included messaging services such as WhatsApp and printed news articles. Rumors were rapidly spread through mobile instant messages, which required a micro-level approach against misinformation as opposed to a macro-level targeting legacy media (television and radio broadcasters, newspapers, and magazines) [24,25].

Impact of Misinformation

Three articles described the impact of misinformation, and two studies demonstrated the repercussions of healthcare advice and stigma [8,26]. Individuals who were highly engaged in social media platforms and believed in conspiracy theories were less likely to wear masks even with health officials recommending it [8]. With increased false claims on vaccination safety on social media, vaccine hesitancy also increased in those who believed conspiracy theories. It was found that 14.8% of the US population who participated in a study on stigma communication about COVID-19 on Twitter in the early stage of the outbreak believed that the pharmaceutical industry created the virus and 28.3% believed the Chinese government created the virus as a bioweapon [26]. There was a positive relationship between conservative and social media and belief in conspiracy theories, whereas mainstream print had a negative relationship with conspiracy theories as did income, white racial identity, and education [8,26].

With regard to vaccination, the perceived threat to the participant and the United States contributed to the association between vaccine intention and conspiracy beliefs, but the belief that the mumps, measles, and rubella (MMR) vaccine is harmful was a strong contributor to vaccine hesitancy [8]. Vaccine intention was directly affected by conspiracy beliefs and indirectly by MMR perception of harm [8]. There was a positive relationship between reliance on mainstream television and vaccination, as well as reliance on social media and perception of MMR harm [8]. Analysis on Twitter aimed to identify the relationship between the presence of misinformation and conspiracy theories in COVID-19-related tweets with the stigmatization of the Asian population. From 155,353 unique COVID-19-related tweets posted between December 31, 2019, and March 13, 2020, more than half of the tweets fell in the classification of peril, which the researchers defined as information that links the stigmatized individual to threats such as physical or social danger [26]. Peril alone does not represent stigma; however, when complemented with other classifications, it indicated stigmatization of the Asian population in relationship to COVID-19. In association with the Asian population, the conspiracy theories labeled COVID-19 as “Wuhan/Chinese Virus” [26].

Strategies to Limit Misinformation

More than half of the articles (11 of 20) examined strategies that could be employed to limit the spread of misinformation. There was discordance between studies regarding whose responsibility it was to combat misinformation spread throughout social media. Two studies found an individual with a sizable social media following to have an impact on denouncing misinformation [11,17]. Other studies concluded those who had a professional responsibility to combat misinformation should take ownership, namely, internal health agencies, scientists, health information professionals, and journalists [4,8,13]. Another study posited that support by conservative parties in these professions could confront conspiracy beliefs surrounding COVID-19 [8].

Four of the eleven articles analyzed how to intervene in misinformation. A study summarizing the World Health Organization Technical Consultation suggested that interventions should be based on science and evidence [27]. A content analysis of twitter hashtags proposed that interventions involving multiple social media platforms are essential to disseminate reliable and vetted information [28]. Ahmed et al., on the other hand, proposed the fight against misinformation should take place on the platform that it arises on [11]. When misinformation was identified, taking down accounts set up to spread misinformation was beneficial and tagging misinformation as going against public health officials provided heightened transparency compared to tagging misinformation as harmful [11,29]. Based on the wide variety of findings, a multifaceted approach should be investigated.

Some studies in the review explored the topic of actionable items that could be imposed on the public to limit the spread of misinformation. One such suggestion was to implement a standard for validating professional status that could be displayed on social media to relay to the public the credibility of the information being presented [30]. Research has shown that an “accuracy nudge” intervention, which involved participants in assessing the accuracy of the news headline, was an effective way of preventing users on social media from spreading misinformation [31]. Both interventions demonstrated the utility of imposing additional scrutiny on the public’s consumption of information.

More than one-fourth (four of 11) of the articles discussed preventative measures that could be taken to prevent misinformation from arising. For instance, a study in Zimbabwe found citizens were most likely to trust an international organization; therefore, the National Association of County and City Health Officials encouraged local health departments to use social media to effectively disseminate evidence-based information to their local constituents [32,33]. Two studies encouraged strategic partnerships to be formed across all sectors to strengthen the analysis and amplification of science-based information [13,27]. Two studies encouraged translating this information into actionable behavior-change messages that should be presented in a culturally specific and community-targeted way to disseminate information to the public [27,32]. All these articles highlight the importance of proactive behavior in preventing the distribution of misinformation.

Discussion

The purpose of this study was to identify the sources and impact of COVID-19 misinformation on social media and examine potential strategies for limiting the spread of misinformation. From this review, three themes emerged: sources of misinformation, the impact of misinformation, and strategies to limit misinformation regarding COVID-19 on social media.

While the COVID-19 pandemic has highlighted numerous vulnerabilities in our public health system and social infrastructure, it is likely that issues of misinformation will be confronted in future public health crises. This study utilized a scoping review methodology to serve as a basis for formulating interventions for combatting COVID-19 misinformation on social media and preventing misinformation in the future.

Sources of misinformation discussed in the articles included Twitter, YouTube, Facebook, and websites dedicated to the dissemination of COVID-19 misinformation, along with mobile instant messaging and others [4,14,21]. Some studies described different sources of misinformation by identifying components such as percentages of both false and factual reports, number of views per source (individual accounts or entire platforms), and political policy alignment [8,26]. Other studies analyzed different forms of misinformation, such as rumors, stigmas, and conspiracy theories, along with factors and underlying context that led to their spread [8,26]. Each with their unique approach, all 20 articles demonstrated the complexity of identifying and analyzing different sources of misinformation related to COVID-19 in today’s vast social media realm. With an ever-growing number of social media platforms, misinformation has more potential than ever to be accessed by a wide range of users. Identifying such sources of misinformation would be a critical step in battling future public health threats.

With countless routes of spread, misinformation about COVID-19 has impacted public health efforts [6,8,26,34]. Dissemination of misinformation and conspiracy theories about COVID-19 via social media was found to be associated with a decrease in perceived pandemic threat, mask-wearing, and trust in COVID-19 vaccines [8]. Individuals responsible for sharing misinformation on social media displayed higher rates of addiction to social media, fatigue from accessing social media, and reluctance to follow COVID-19 public health recommendations [6]. Similarly, believers of COVID-19-related conspiracy theories were less likely to participate in preventative measures, including mask-wearing and vaccination against COVID-19 [8]. Beyond the effects of misinformation on individuals and public health efforts, studies demonstrated an increase in the stigmatization of Chinese individuals associated with social media postings blaming them for the pandemic or referring to COVID-19 as the “Wuhan or Chinese virus” [26]. Misinformation is certainly not novel, but social media has catapulted it to new heights. If proper measures are not taken, it will continue to impede future public health efforts and cause detrimental effects on the infrastructure of healthcare and society [34].

Many of the studies in the review investigated various strategies that may be used to limit the spread of misinformation. Among these, a variety attempted to identify whose responsibility it should be to combat misinformation. Some reports suggested those with professional obligations, such as health professionals, scientists, and local health departments, should take a leading role in combating misinformation [34]. Other sources demonstrated that public figures with a large following had a significant impact on denouncing misinformation [34]. Another common consideration of how misinformation was received largely depended on how it was tagged (i.e., as harmful vs. going against public health officials) [11,28,29,30]. By collectively investigating different components, this scoping review has highlighted potential strategies for limiting the spread of misinformation related to COVID-19 [34]. Considering these different components, such as who should combat misinformation and methods to do so, will aid in curtailing the spread of misinformation in the future when encountering future pandemics or health threats [11,28,29,30].

Various approaches were used to identify, describe, and analyze misinformation on social media related to COVID-19. The many types of data collected across the studies were likely a result of the underlying complexity of social media and the different forms it can take. Despite having different study and analysis designs, the included articles contributed to overarching themes.

Limitations of included studies

With a topic as evolving as COVID-19 in 2020, the resulting articles in this review had their limitations in accurately representing the presently available data regarding misinformation on social media related to the virus. When considering a global pandemic, language restrictions (most commonly, English-only studies) may limit the generalizability of such studies to the public as semantics and context may get lost in translation. Another limitation in several of the study designs was the exclusion of potentially relevant data, while other limitations included small sample sizes and specific identifiers such as politics and religion. Additionally, the limit in search time frames, namely, cross-sectional studies, during a rapidly changing pandemic may have led to missed topics and important messages. Lastly, data from the included studies were subject to biases due to misclassification of qualitative data, self-reported data, the tendency of younger populations to use social media, and confounding variables such as media literacy or cognitive sophistication.

Limitations of the review process

First, there was a relatively narrow timeframe used to search for articles. As the course of the COVID-19 pandemic has undulated with the emergence of new strains, a search for articles within a distinct timeframe may not accurately represent the course of the COVID-19 pandemic, which is rapidly changing. Additionally, this review only included articles written in English, which may not be representative of the global pandemic. Lastly, relevant articles may have been excluded due to strict inclusion and exclusion criteria.

Considerations for future research

More research is needed to investigate methods used to combat misinformation to compare their efficacy and/or compare sources of education aimed to combat misinformation. Studies should also examine the role of misinformation in other public health crises, such as the Ebola virus epidemic or the anti-vaccination movement. Paralleling these findings with the results of this scoping review may reveal useful strategies for addressing misinformation regarding future public health crises.

## Conclusions

Social media creates an easy, accessible outlet for the dissemination of misinformation and disinformation about the COVID-19 pandemic due to a lack of regulation of content. The spread of misinformation has been shown to create anxiety and doubt surrounding public health official advice. The results of this review may help us better understand the effect that COVID-19 misinformation on social media has on a population’s thoughts, beliefs, and actions. Sources of misinformation about COVID-19 included Twitter, YouTube, and Facebook. Dissemination of misinformation about COVID-19 via social media was found to be associated with widespread negative outcomes such as higher rates of addiction to social media, fatigue from accessing social media, and reluctance to follow COVID-19 public health recommendations. The studies also demonstrated an increase in stigmatization against certain Asian populations brought about by blaming this group for the pandemic. Findings of this review highlight the sources and impact of misinformation on social media related to COVID-19 along with potential strategies to mitigate misinformation. Additionally, it may help guide future efforts in the fight against COVID-19 as well as other public health threats.
